# Gut microbiota composition and changes in patients with sepsis: potential markers for predicting survival

**DOI:** 10.1186/s12866-024-03188-6

**Published:** 2024-02-01

**Authors:** Feiyu Luan, Yang Zhou, Xiaohui Ma, Yue Li, Yahui Peng, Xiaonan Jia, Nana Li, Xibo Wang, Yinghao Luo, Mingyin Man, Qianqian Zhang, Chunying Wang, Kaijiang Yu, Mingyan Zhao, Changsong Wang

**Affiliations:** 1 Departments of Critical Care Medicine, the First Affiliated Hospital of Harbin Medical University, Harbin Medical University, Harbin, 150001 Heilongjiang China; 2 Departments of Critical Care Medicine, the Second Affiliated Hospital of Harbin Medical University, Harbin Medical University, Harbin, 150081 Heilongjiang China

**Keywords:** Sepsis, Gut microbiota, Mortality, Intensive care, Outcome, Prediction

## Abstract

**Background:**

Sepsis can cause immune dysregulation and multiple organ failure in patients and eventually lead to death. The gut microbiota has demonstrated its precise therapeutic potential in the treatment of various diseases. This study aimed to discuss the structural changes of the gut microbiota in patients with sepsis and to analyze the differences in the gut microbiota of patients with different prognoses.

**Methods:**

We conducted a multicenter study in which rectal swab specimens were collected on the first and third days of sepsis diagnosis. A total of 70 specimens were collected, and gut microbiota information was obtained by 16S rRNA analysis.

**Results:**

The relative abundance of Enterococcus decreased in rectal swab specimens during the first three days of diagnosis in patients with sepsis, while the relative abundance of inflammation-associated Bacillus species such as *Escherichia coli*, Enterobacteriaceae, and Bacteroidetes increased. By comparing the differences in the flora of the survival group and the death group, we found that the abundance of Veillonella and Ruminococcus in the death group showed an increasing trend (*p* < 0.05), while the abundance of Prevotella_6 and Prevotella_sp_S4_BM14 was increased in surviving patients (*p* < 0.05).

**Conclusions:**

The Firmicutes/Bacteroidetes ratio, reflecting overall gut microbial composition, was significantly lower on day three of sepsis diagnosis. Changes in the abundance of specific gut microbiota may serve as prognostic markers in patients with sepsis.

## Introduction

There are 100 trillion parasitic microorganisms in the human gastrointestinal (GI) tract [1], including bacteria, viruses, fungi, archaea, and protozoa, which are collectively termed the gut microbiota [2]. Research has long focused on the pathogenicity of gut microbiota, but new studies suggest that the gut microbiota is a complex ecosystem that plays an important role in many aspects of host metabolism, immunity and health [3, 4]. Perturbations of the gut microbiota even during infancy can affect growth, development and health [5].

Sepsis is a serious global health problem and the most common cause of death in hospitals [6]; it can lead to immune dysregulation and multiple organ failure, causing 11 million deaths worldwide every year [7–9]. Over the past few decades, in critical illness, the gut has been considered the driver of associated infectious complications [10]. However,current studies have found that the gastrointestinal tract (GIT) is the largest immune organ and maintains systemic immune homeostasis [11]. Patients with sepsis suffer from both sustained excessive inflammation and immune suppression [12], and the gut microbiota and its associated metabolites play an essential role in regulating the host immune response to infection [13].

The gut microbiota has enormous genetic and metabolic diversity, and it has fully demonstrated promise in precision medicine and personalized therapy in recent years [14]. The relationship between intestinal gut microbiota and sepsis has become a hot field [15]. Increasing evidence shows that gastrointestinal dysfunction is associated with high mortality in patients with sepsis [16, 17]. Necessary medical treatment of sepsis patients leads to the collapse of gut microbiota diversity [18], and the examination and management of the gut microbiota in patients with sepsis has become an important part of clinical treatment [19], Therefore, understanding the changes of gut microbiota in sepsis patients has become an urgent problem to be solved.

In this study, we continuously collected rectal swabs from sepsis patients on the first and third day after diagnosis, and tried to describe the characteristics of gut microbiota in sepsis patients by dynamic observation and combining with the prognosis and clinical factors of patients, so as to determine the relationship between gut microbiota and the survival rate of patients, and the relationship between the changes of gut microbiota and the development of sepsis. We attempted to elucidate the correlation of gut microbiota with age, lactate, procalcitonin, mean arterial pressure (MAP) and other factors.

## Methods

### Subjects and inclusion criteria

This was a multicenter study with patients from the ICU wards of the First Affiliated Clinical Medical College of Harbin Medical University and the Second Affiliated Clinical Medical College of Harbin Medical University. The inclusion criteria are in line with the definition of sepsis and septic shock in the third international consensus [20]. Patients younger than 18 years old, pregnant, with IBD or terminal diseases, and patients undergoing colostomy or ileostomy were excluded. In the presence of clinicians, patients’ families were informed of the study and signed informed consent forms. In this study,all research was conducted in accordance with the Declaration of Helsinki,all patients’ privacy rights are respected. A total of 70 clinical specimens from 41 sepsis patients were collected during a six-month period from March to September 2021.

### Specimen collection and preservation

For patients who met the inclusion criteria, specimens were collected on the first and third days of sepsis diagnosis using sealed RNase- and DNase-free cotton swabs, which were then placed in protective tubes made of polystyrene. With the patient in the left lateral decubitus position, a rectal swab was inserted 2-3 cm into the rectal sphincter, rotated 360°, removed and checked for successful stool collection. Immediately after successful collection, the specimens were transferred and placed in a − 80 °C freezer until DNA extraction.

### Data records

We collected clinical data through electronic medical records, including sex, age,PH,heart rate(HR),sequential organ failure assessment (SOFA) score, physiology and chronic health (APACHE) II score, blood lactate content(LAC), procalcitonin(PCT) level, ICU length of stay, systolic and diastolic blood pressure(SBP,DBP), and we calculated the mean arterial pressure(MAP).After one month, follow-up was performed to record the mortality of patients on the 7th and 28th days after diagnosis to complete the evaluation.

### 16SrRNA

Extract the DNA in the specimen and use NanoDropOne to detect the concentration and purity, and use the primers with barcode for PCR amplification. Amplicons were sequenced on the MiSeq platform.The sequence clustering algorithm was used to cluster sequences with a similarity of more than 97% to generate an OTU table. The specimens were analyzed for alpha and beta diversity based on the OTU table.

### Statistical analysis

The demographic characteristics of patients were assessed using descriptive statistics. Using the grouped case–control method, 70 specimens were grouped and compared according to the time of specimen collection and the survival time of patients. The t test was used for those that conformed to the normal distribution, the rank-sum test was used for those that did not conform to the normal distribution, and the diversity of different groups was analyzed. The z value was obtained by normalizing the relative abundance of the species (the difference between the relative abundance of this sample in this taxonomy and the average relative abundance of all specimens in this taxonomy divided by the standard deviation of all specimens in this taxonomy). The obtained values are displayed in the Species Abundance Analysis Graph. The relative abundance of all OTUs in different specimens was counted, the dissimilarity coefficient matrix between different specimens was calculated using the selected distance formula, and the matrix was hierarchically clustered to obtain the correlation map between gut microbiota and specific factors (SOFA score, ICU length of stay, pH, age, heart rate, procalcitonin, systolic blood pressure, diastolic blood pressure, mean arterial pressure and blood lactate). In the LEfSe analysis, the nonparametric Kruskal–Wallis rank-sum was first used to detect the abundance differences between different groups, and then the Wilcoxon rank-sum was used to test the difference consistency of the different substances in different subgroups between groups. Finally, linear discreminant analysis (LDA) was used to estimate the size of the effect of each microbiota on the difference. All tests used *p* = 0.05 as the threshold for significance.

## Results

### Study cohort

We obtained 70 specimens from 41 sepsis patients. Patient demographics and clinical characteristics are reported in Table 1.

**Table 1:** Demographics and clinical characteristics of study cohort

Characteristic	Study cohort (*n* = 41)
Mean age (SD), y	62.49(15.22)
Sex(Male/Female)	19/22
Admission type	
Surgical (emergency)	11
Surgical (elective)	1
Mean SOFA (SD)	8.54(2.88)
Mean APACHE II (SD)	18.63(5.76)
Mean ICU length of stay, days (SD)	7.17(4.84)
Nutrition way	
Enteral nutrition	20
Parenteral nutrition	21
Infection site	
Lung	18
Abdomen	18
Urinary system	5
7-day mortality	9.76%
28-day mortality	17.07%

### Analysis of differences in the gut micro supplementarybiota between the first and third days of sepsis

First, according to the time of specimen collection, we divided 70 specimens into the SepsisD1 group (35 specimens collected on the first day of diagnosis of sepsis) and SepsisD3 group (35 specimens collected on the third day after the diagnosis of sepsis). The core group of gut microbiota, microbial diversity and community structure of the specimens were preliminarily assessed.

### Core group of gut microbiota

**Fig. 1:**
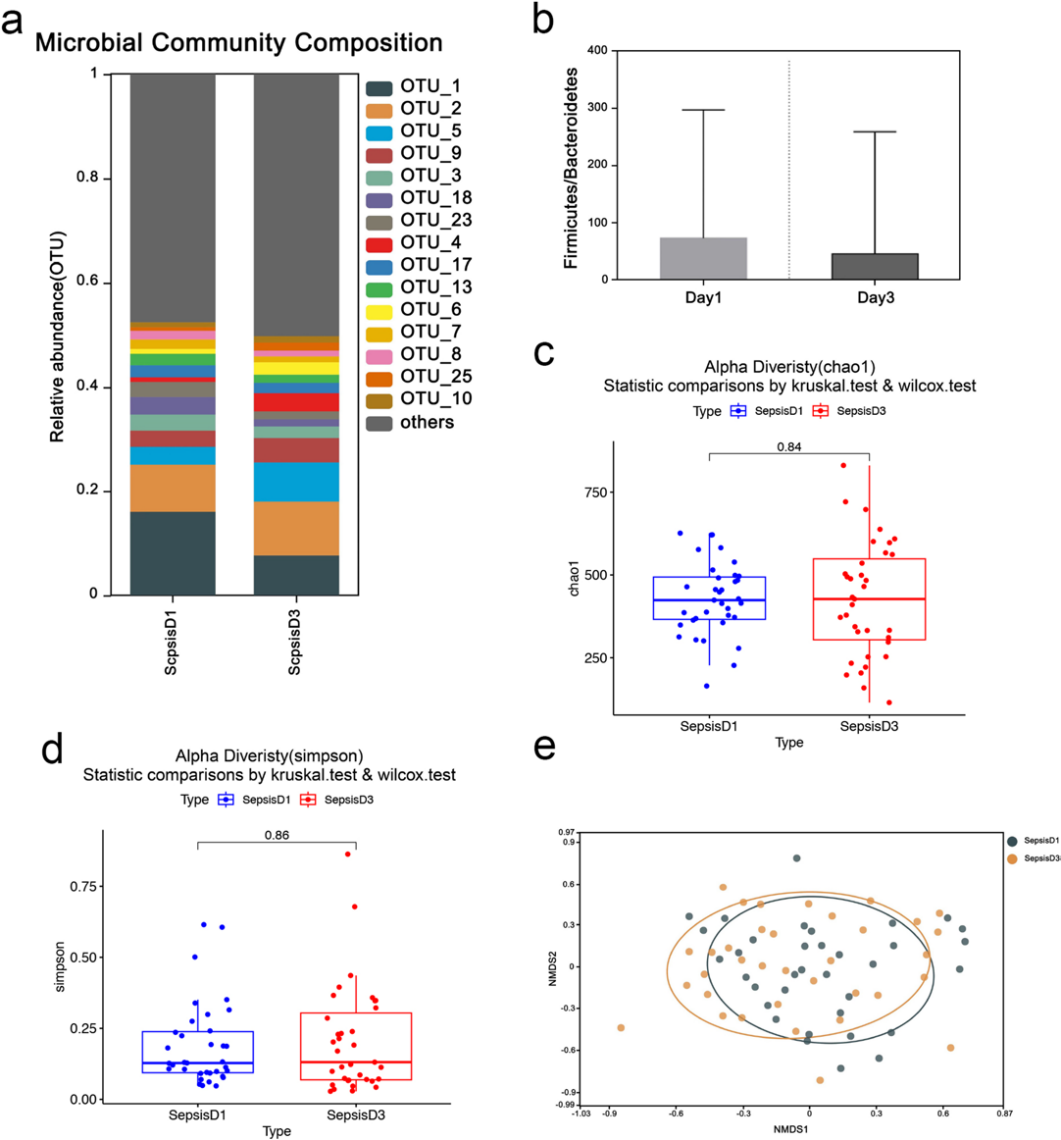
**a** Histogram of gut microbiota composition. **b-c** The alpha diversity of sepsisD1 and sepsisD3 groups was analyzed, and the **b** Chao1 and **c** Simpson index of the samples were evaluated.**d** The NMDS analysis chart shows the β diversity analysis of sepsisd 1 and sepsisD3. **e** Comparison of the Firmicutes/Bacteroidetes ratio

Calculate the relative abundance of bacteria in each sample; OTUs with relative abundance over 1% were selected to draw the relative abundance distribution map of top 15 intestinal microflora in SepsisD1 group and SepsisD3 group.(Fig. 1a). We can see that the core genus of sepsis patients is *Enterococcus* (OTU1), *Escherichia-Shigella* (OTU2), *Enterobacteriaceae* (OTU5), *Bacteroides* (OTU9, OTU13), *Corynebacterium* (OTU3), *Porphyromonas* (OTU18), *Anaerococcus* (OTU23), *Acinetobacter* (OTU4), *Staphylococcus* (OTU17), *Campylobacter* (OTU6), etc. Compared with the sepsisD1 group, we found that the relative abundance of *Enterococcus* was decreased in the sepsisD3 group, and the relative abundance of *Escherichia-Shigella*, *Enterobacteriaceae* and *Bacteroides* was increased. The Firmicutes/Bacteroidetes ratio, reflecting overall gut microbial composition, was significantly lower on day three of sepsis diagnosis(Fig. 1e).

### Alpha diversity analysis

The alpha diversity index was analyzed for the SepsisD1 group and SepsisD3 group of specimens (Fig. 1b,c), and the Chao-1 index and the Simpson index were used for quantification. There was no significant difference in the diversity index between the sepsisD1 and sepsisD3 groups (*p* = 0.83).

### Beta diversity analysis

Afterward, beta diversity analysis was performed on the SepsisD1 group and SepsisD3 group of specimens, and the spatial location map of the specimens was obtained by NMDS (Fitness Multi-Microscale Method) (Fig. 1d). The close spatial distance between the sepsisD1 and sepsisD3 specimens indicated that the gut microbiota did not undergo drastic changes in species composition during the first three days of sepsis diagnosis.

### Gut microbiota and factors

**Fig. 2:**
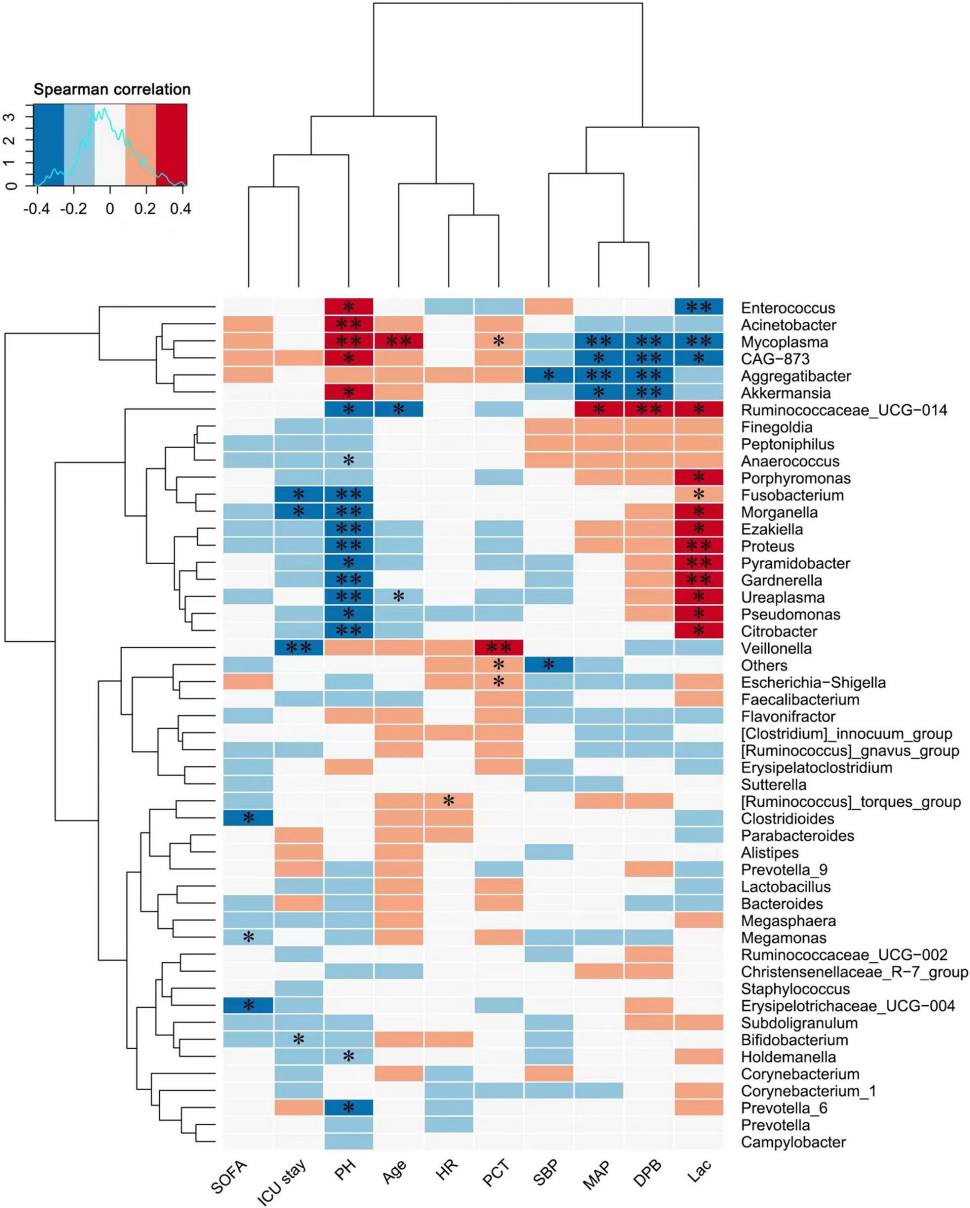
Heatmap of the beta diversity matrix. *means *P* < 0.05, **means *P* < 0.01

We selected 10 factors, including the SOFA score, ICU length of stay, PH, age, HR, PCT, SBP,DBP, MAP and LAC. The distance between specimens was used to obtain the beta diversity matrix, which is displayed by a heatmap (Fig. 2).

Through the heatmap, we found that *Ezakiella*, *Proteus*, *Pyramidobacter*, *Gardnerella*, *Ureaplasma*, *Pseudomonas* and *Citrobacter* microorganisms were proportional to the blood lactate value and inversely proportional to the pH value (*p* < 0.05). Among them, *Proteus*, *Pyramidobacter*, *Gardnerella* and blood lactate were more significantly related (*p* < 0.01). *Aggregatibacter*, *Mycoplasma*, *CAG-873*, and *Akkermansia* were inversely proportional to patients’ systolic blood pressure, diastolic blood pressure and mean arterial pressure, and diastolic blood pressure was significantly affected (*p* < 0.01). *Veillonella* was proportional to PCT (*p* < 0.01) and inversely proportional to the average length of stay in the ICU of patients. *Mycoplasma* was the only factor closely related to age (*p* < 0.01) and was inversely proportional to diastolic blood pressure, mean arterial pressure and pH.

### Analysis of differences in gut microbiota between survivors and deceased patients

To study the relationship between the prognosis of patients with sepsis and the gut microbiota, the 7-day and 28-day survival rates of the patients were calculated, and the patient specimens were divided into the following groups according to the survival rate: Live7D1 (specimens from 7-day survivors on the first day of diagnosis), Death7D1 (specimens from 7-day deceased patients on the first day of diagnosis), Live7D3 (specimens from 7-day survivors on the third day after the diagnosis), Death7D3 (specimens from 7-day deceased patients on the third day after the diagnosis), Live28D1 (specimens from 28-day survivors on the first day of diagnosis), Death28D1 (specimens from 28-day deceased patients on the first day of diagnosis), Live28D3 (specimens from 28-day survivors on the third day after the diagnosis), and Death28D3 (specimens from 28-day deceased patients on the third day after the diagnosis). Furthermore, the community structure and species differences of the specimens were analyzed to determine the differential microbiota related to the patient prognosis.

### Species community structure analysis

**Fig. 3:**
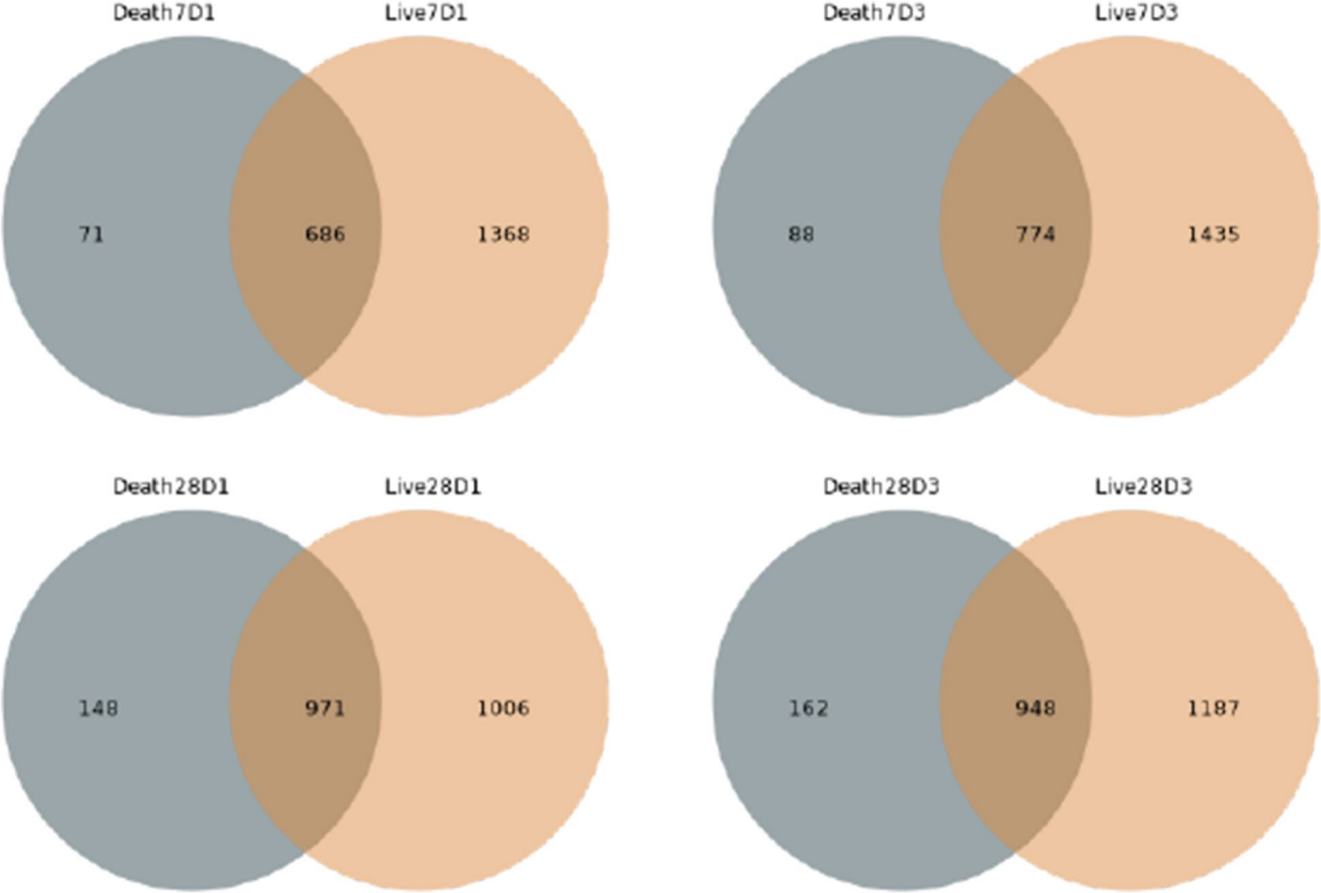
Venn diagram shows the number of common and unique OTUs in different groups

First, we counted the occurrence of OTUs in the grouping, and by plotting the Venn diagram to show the number of common and unique OTUs, we found that the patients in the death group had fewer types of OTUs and fewer unique OTUs (Fig. 3).

### Core group composition analysis

The top 15 species in the SepsisD1 group and SepsisD3 group of specimens were selected, the relative abundance was compared, and the differences in dominant species between the two groups were analyzed.

**Fig. 4:**
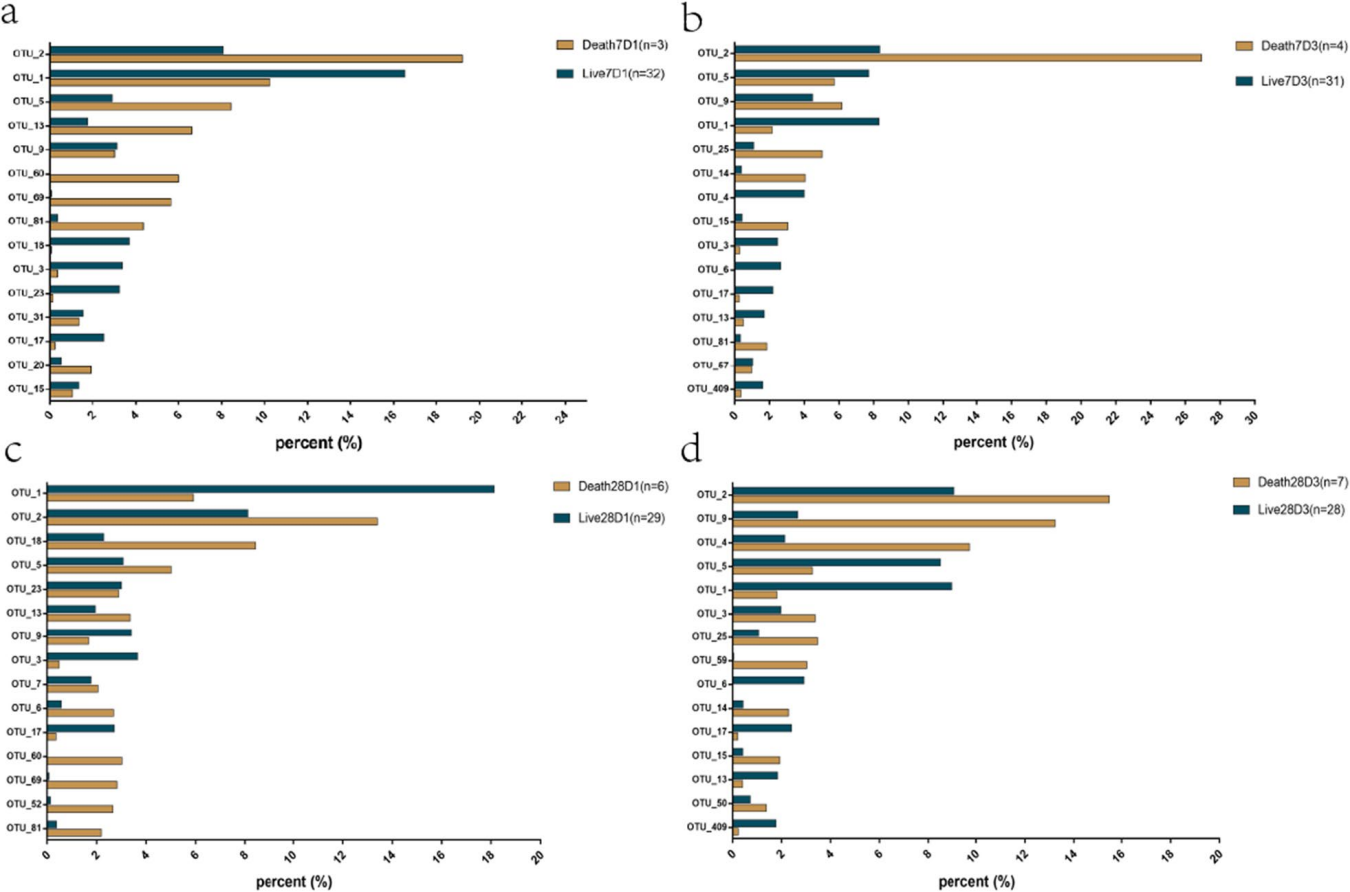
**a-b** According to 7-day survival/death, the relative abundance of the top 15 OTU in **a** SepsisD1 and **b** SepsisD3 groups. **c-d** According to 28-day survival/death, the relative abundance of the top 15 OTU in **c** SepsisD1 and **d** SepsisD3 groups

Comparing the Live7D1 and Death7D1 groups (Fig. 4a), the relative abundance of OTU_2 (g_Escherichia-Shigella), OTU_60 (g_Aggregatibacter), OTU_69 (g_Fusobacterium), and OTU_81 (g_Veillonella) was higher in deceased patient specimens.

Comparing the Live7D3 and Death7D3 groups (Fig. 4b), the relative abundance of Escherichia-Shigella (OTU2) remained higher in the death group, and the relative abundance of OTU14 and OTU15 was also higher in the death group. It is worth noting that these OTUs come from the same genus, *Ruminococcus*, and we found no significant difference in OTU15 between surviving and deceased patients on the first day of diagnosis.

Comparing the Live28D1 and Death28D1 groups, *Porphyromonas* (OTU18), *Aggregatibacter* (OTU60), *Fusobacterium* (OTU69), *Corynebacterium* (OTU52), and *Veillonella* (OTU81) were more abundant in the specimens of deceased patients (Fig. 4c).

Comparing the Live28D3 and Death28D3 groups, *Escherichia-Shigella* (OTU2), *Bacteroides* (OTU9), *Acinetobacter* (OTU4), *Pyramidobacter* (OTU59), and *Ruminococcus* (OTU15) were more abundant in the specimens of deceased patients (Fig. 4d).

### Species difference analysis

**Fig. 5:**
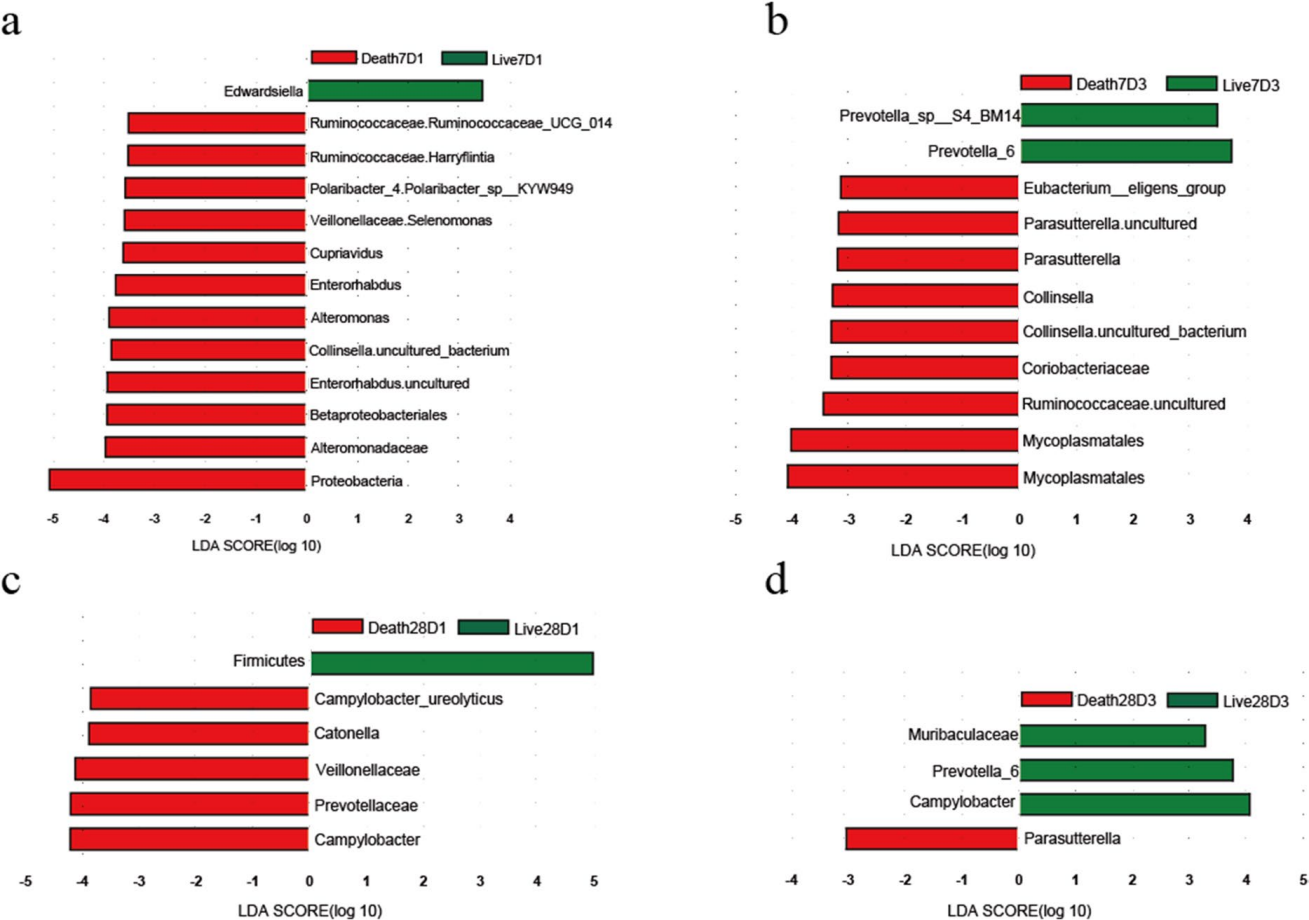
The LDA graph represents species with significantly different abundances in different groups when the LDA value was greater than the set value. **a-b** According to 7-day survival/death, LDA value of gut microbes in **a** SepsisD1 and **b** SepsisD3 groups. **c-d** According to 28-day survival/death, LDA value of gut microbes in **c** SepsisD1 and **d** SepsisD3 groups

Linear discriminant analysis effect size (LEfSe) was used to estimate the effect size of each microbiota on the difference in patient survival/death. Comparing the Live7D1 and Death7D1 groups (Fig. 5a), 12 bacterial groups were found to be associated with patient death, of which *Veillonella* was among the top 15 most abundant. Comparing the Live7D3 and Death7D3 groups (Fig. 5b), it was found that 9 bacterial groups were associated with death, and *Prevotella_sp_S4_BM14* and *Prevotella_6* were associated with survival. we also found that, whether comparing the specimens from the first day of sepsis diagnosis or the third day of the diagnosis of sepsis, *Ruminococcaceae* was associated with patient death.

Comparing the Live28D1 and Death28D1 groups (Fig. 5c), we found that 5 bacterial groups were associated with patient death. Comparing the Live28D3 and Death28D3 groups (Fig. 5d), we found that 3 bacterial groups were associated with survival, and 1 bacterial group was associated with death. Here we also found that *Prevotella_6* is associated with patient survival.

### Changes in gut microbiota in patients with different prognoses

To observe the relationship between the changes in gut microbiota and the prognosis of patients, the top 30 genera of relative abundance were selected for cluster analysis, and the different changing trends of the gut microbiota of the patients who survived or died were observed.

**Fig. 6:**
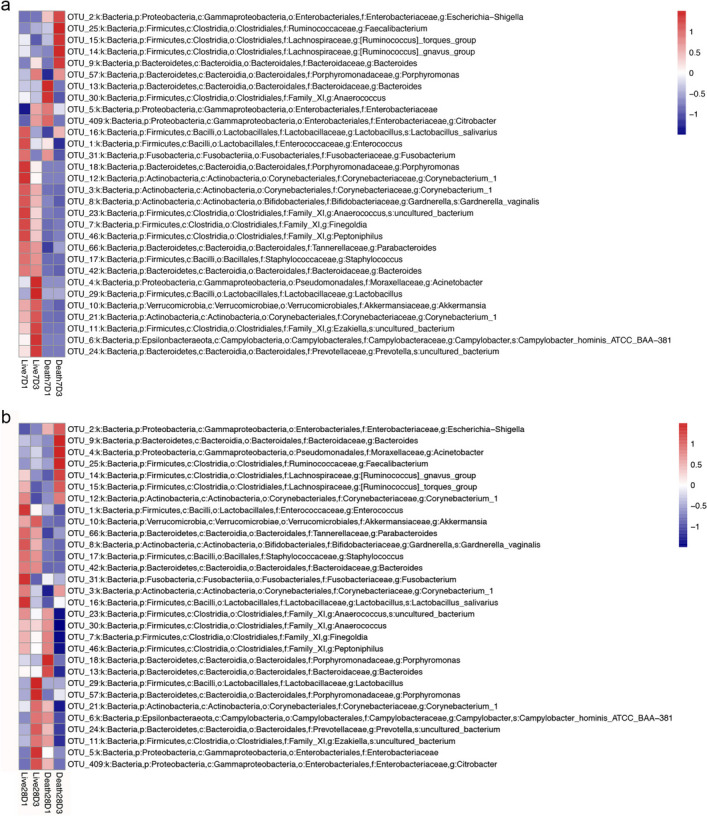
According to the survival/death of **a** 7-day and **b** 28-day, the relative abundance changes of gut microbes in sepsis patients were displayed by using species abundance cluster analysis diagram

Grouped according to the 7-day survival rate (Fig. 6a), compared with the Live7D1 group, the abundance of *Porphyromonas* (OTU18), *Corynebacterium-1*(OTU12), *Gardnerella*(OTU8), *Anaerobicococcus*(OTU23), *Finegoldia*(OTU7), *Peptoniphilus*(OTU46), *Parabacteroides*(OTU66), *Staphylococcus*(OTU17) and *Bacteroides*(OTU42) was lower in the Live7D3 group, and *Acinetobacter*(OTU4), *Lactobacillus*(OTU29), *Akkermansia*(OTU10), *Corynebacterium* (OTU21), *Ezakiella*(OTU11), *Campylobacter*(OTU6) and *Prevotella*(OTU24) were more abundant. Compared with the Death7D1 group, *Anaerococcus*(OTU30), *Enterobacteriaceae*(OTU5) and *Citrobacter*(OTU40) were less abundant in the Death7D3 group, and *Escherichia-Shigella*(OTU2),*Faecalibacterium*(OTU25), *Ruminococcus*(OTU15) *torques*, *Ruminococcus gnavus*(OTU14) and *Porphyromonas*(OTU57) were more abundant. *Faecalibacterium*, *Ruminococcus torques* and *Ruminococcus gnavus* decreased in abundance in the survival group and increased in abundance in the death group.

Grouped according to 28-day survival(Fig. 6b), compared with the Live28D1 group, *Enterococcus*(OTU1), *Akkermansia*(OTU10), *Parabacteroides*(OTU66), *Gardnerella*(OTU8), *Staphylococcus*(OTU17), *Bacteroides*(OTU42) and *Fusobacterium*(OTU31) were less abundant in the Live28D3 group, and *Lactobacillus*(OTU29), *Porphyromonas*(OTU57), *Corynebacterium* (OTU21),*Campylobacter*(OTU6), *Prevotella*(OTU24), *Ezakiella*(OTU11), *Enterobacteriaceae*(OTU5) and *Citrobacter*(OTU409) were more abundant.Compared with the Death28D1 group, *Anaerococcus*, *Finegoldia*, *Peptoniphilus* and *Porphyromonas* were less abundant in the Death28D3 group, and *Escherichia*, *Acinetobacter*, *Faecalibacterium*, and *Ruminococcus* were more abundant. The abundance of *Campylobacter*, *Prevotella* and *Ezakiella* increased gradually in the survival group and decreased in the death group.

## Discussion

Dysregulation of gut microbiota is associated with a variety of digestive diseases, such as irritable bowel syndrome (IBS), inflammatory bowel disease, colorectal cancer (CRC), liver disease, and pancreatic disease [21–24]. At present, the help of the gut microbiota in the diagnosis and treatment of diseases is not limited to intestinal diseases. In diseases such as type 2 diabetes, Alzheimer’s disease, and cardiovascular and cerebrovascular diseases, the gut microbiota also shows great prospects for biological treatment [22–27]. However, there are still few relevant studies on the gut microbiota in patients with sepsis. By recording the dynamic changes of the gut microbiota in patients with sepsis, we correlated the changes in the gut microbiota in patients with sepsis with patient prognosis. Through differential analysis, three bacteria groups related to the prognosis of sepsis were summarized, and there are few studies on the relationship between these bacteria groups and sepsis at present.

### Veillonella

In the heatmap of environmental factors, we found that PCT was proportional to the *Veillonella* microbiota (OTU81 Veillonella) (*p* < 0.01). Comparing the top 15 microbiota between 7-day surviving and deceased patients, we also found the content of *Veillonella* was higher in the specimens of the deceased patients. The LEfSe analysis predicted that patient mortality was associated with *Veillonellaceae* (*p* < 0.05), a lactic acid-fermenting bacterium that colonizes the oral, genitourinary, respiratory, and gut microbiomes found in healthy humans. *Veillonella* is usually found in the mouth [28]. Loomba R et al. found that *Veillonella* is associated with bile acid metabolism and has the potential to be a marker for predicting efficacy in the treatment of primary sclerosing cholangitis, nonalcoholic fatty liver disease and other diseases [29]. At the same time, some studies have found that the dominant bacteria in the upper gastrointestinal tract, *Veillonella*, is almost absent in the lower gastrointestinal tract, and that rectal swabs of deceased patients have higher levels of *Veillonella*. The abnormal migration of microbiota is likely to predict relatively poor clinical outcomes.

### Ruminococcus

When comparing the differences among the top 15 microbiota in the specimens of 7-day surviving/deceased patients on the third day after the diagnosis, we found that OTU14 and OTU15 were higher in the specimens of the deceased patients. Interestingly, OTU14 and OTU15 both belong to *Ruminococcus*. In the LEfSe analysis, we found that *Ruminococcaceae* had a worse effect on the prognosis of patients by comparing the specimens of patients with different prognoses at 7 days (*p* < 0.05). *Ruminococcus* is a Gram-positive anaerobic coccus [30]. Enrique et al. found a transient, dramatic increase in *Ruminococcus* abundance that corresponds to increased disease activity in patients with inflammatory bowel disease [31]. There are also multiple reports linking *Ruminococcus* with activity in Crohn’s disease and other immune diseases [32–35]. Matthew et al. found that *Ruminococcus* synthesizes and secretes glucorhamnan, which can effectively induce dendritic cells to secrete inflammatory cytokines (TNFα) [36]. In the early stage of sepsis, overactivated Toll-like receptor (TLR) activates intracellular transcription factors, such as NF-κB, and induces the generation and release of proinflammatory cytokines(e.g., interferon-α [IFN-α], interleukin-6 [IL-6], interleukin-8 [IL-8], and tumor necrosis factor-α [TNF-α]), thus triggering a cytokine storm and leading to the first death peak of sepsis patients [37]. We do not yet know whether *Ruminococcus* affects immune dysregulation in sepsis patients and thus affects prognosis.

### Prevotella

Comparing the changes in the microbiota of the specimens from the first day and the third day of the surviving patients, it was found that the abundance of *Prevotella* increased in the specimens of the surviving patients on the third day. *Prevotella* is a diverse genus of gram-negative anaerobic bacteria [38]. Fifty-seven species of *Prevotella* have been identified [39]. They colonize many parts of the human body and are also the main genus of the three reported human enterotypes. Their relative abundances were negatively correlated with the relative abundance of *Bacteroides* [40]. The higher the diversity of *Prevotella*, the more fermentative the microbiome for the benefit of the human gut. In our results, it was found that *Prevotella_6* and *Prevotella_sp_S4_BM14* were significantly different in the microbiota specimens of the survival group on the third day. It may be that the abundance of *Prevotella_6* and *Prevotella_sp_S4_BM14* in sepsis patients increased on the third day after the diagnosis, indicating a good prognosis.

Unlike the previous study by Gloria M et al. [10], that showed a significant reduction in gut microbial diversity during the ICU stay, We think this may be due to the fact that the second sample in Gloria M et al’s study was collected on the fifth to seventh day after admission to ICU. We can collect samples from sepsis patients admitted to ICU on the 1st, 3rd, 5th and 7th day after diagnosis in the follow-up experiments to verify the changes of intestinal microflora. The imbalance of immune homeostasis is the pathological mechanism of sepsis death. As a broad-spectrum immunomodulator, gut microbiota can regulate the functions of various immune cells in the host, and understand the changes of gut microbiota and the abundance of specific microbiota in sepsis patients, which may provide new ideas for individualized treatment of sepsis patients.

## Conclusion

Compared with the samples on the first day after diagnosis of sepsis, the relative abundance of *Enterococcus* in the samples on the third day after diagnosis decreased, while the relative abundance of *Bacillus* species associated with inflammation, such as *Escherichia-Shigella*, *Enterobacteriaceae* and *Bacteroides*, increased. There was no significant difference in microbial community diversity in the first three days after the diagnosis of sepsis, and the ratio of Firmicutes/Bacteroides reflecting the overall intestinal microbial composition decreased.

Species community structure analysis showed that the patients in the death group had fewer bacterial species, and through the analysis of species composition and species differences, it was found that *Ruminococcus* and *Veillonella* were relatively abundant in the deceased patients and may have a certain impact on poor prognosis (*p* < 0.05), while *Prevotella_6* and *Prevotella_sp_S4_BM14* may predict a good prognosis (*p* < 0.05).

## Limitations

Small sample size of patients in the death group; Some patients failed to have specimens collected on the third day of sepsis due to gastrointestinal dysfunction and total parenteral nutrition support.

## Data Availability

The datasets generated or analyzed during this study areavailable from the corresponding author on reasonable request.

## Abbreviation

GI: gastrointestinal
GIT: Gastrointestinal tract
ICU: Intensive Care Unit
SOFA: Sequential Organ Failure Assessment Score
APACHE II: Physiology And Chronic Health Score
LAC: Blood Lactate Content
PCT: Procalcitonin
SBP: Systolic Blood Pressure
DBP: Diastolic Blood Pressure
MAP: Mean Arterial Pressure
PCR: Polymerase Chain Reaction
OTU: Operational Taxonomic Unit
LEfSe: Linear Discriminant Analysis Effect Size
